# Pancreatoduodenectomy in patient with perforated duodenal diverticulum and peritonitis: Case report

**DOI:** 10.1016/j.ijscr.2019.04.011

**Published:** 2019-04-10

**Authors:** Justus Philip, Andrei Cocieru

**Affiliations:** a Department of Surgery, Summa Akron City Hospital, Akron, Ohio, United Staes; b Northeastern Ohio Medical University, Roostown, Ohio, United Staes

**Keywords:** Pancreatoduodenectoomy, Duodenal diverticulum, Perforation

## Abstract

**Introduction:**

Duodenal diverticula are quite prevalent in general population, seen on up to 5% of radiology studies and up to 22% of autopsy examinations.

**Presentation of the case:**

70 years old female was admitted to the hospital with epigastric pain, fevers and elevated white cell count. Abdominal CT scan demonstrated evidence of perforated duodenal diverticulitis which failed to improve with IV antibiotics. Emergent pancreatoduodenectomy was performed with full recovery and uncomplicated hospital stay.

**Discussions:**

Conservative therapy with antibiotics and bowel rest is successful in majority cases of perforation. Failure of conservative therapy demands surgical management. Variety of surgical approaches ranging from simple diverticulectomy to segmental resection, duodenal exclusion/bypass to pancreatoduodenectomy are available.

**Conclusion:**

Pancreatoduodenectomy is an option when complicated duodenal diverticulum is not resolved with conservative or interventional therapy. This report has been written in concordance with the SCARE criteria Agha et al. [1].

## Highlights

• Duodenal diverticulum is present in 5–22% of population.
• Complicated diverticulum can lead to perforation, bleeding, obstruction, pancreatitis.
• Pancreatoduodenectomy is an option when all other surgical approaches are not usable.

## 1 Introduction

Duodenal diverticula are quite prevalent in general population, seen on up to 5% of radiology studies and up to 22% of autopsy examinations. [2] Only 5% of all diverticula will ever become symptomatic and develop complications. We present a case of surgical management of freely perforated duodenal diverticulum with peritonitis [1].

## 2 Case presentation

**Fig. 1:**
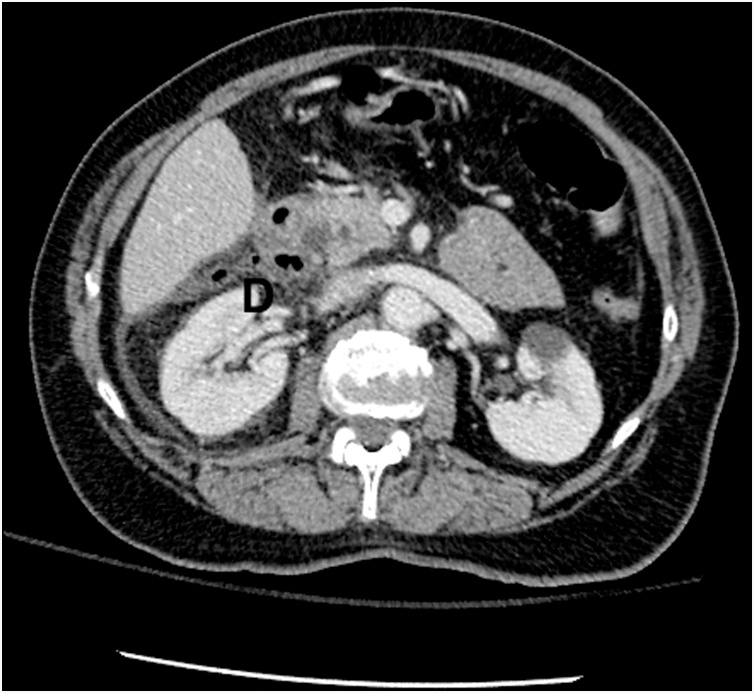
CT scan of perforated duodenal diverticulum marked with letter D.

**Fig. 2:**
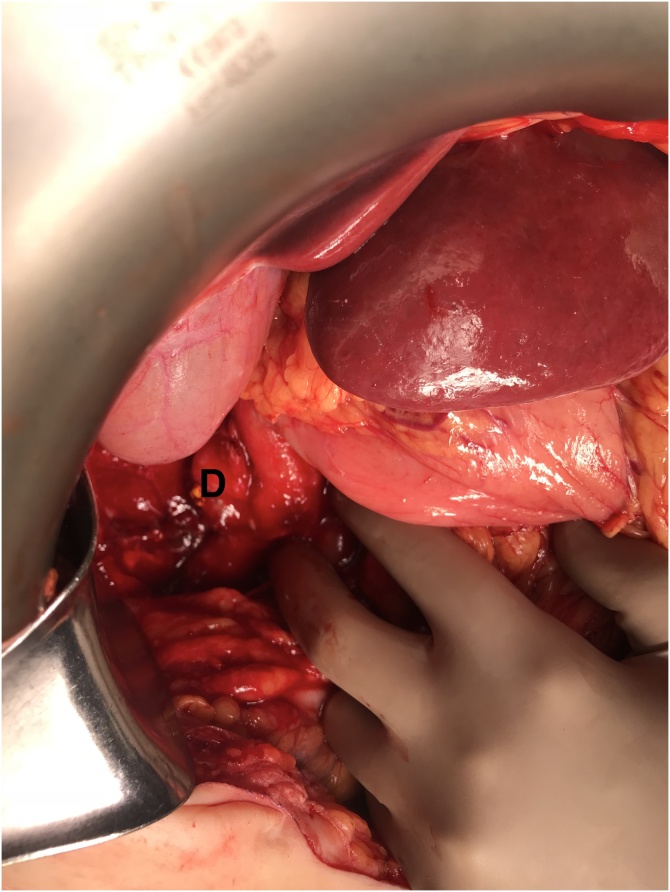
Intraoperative photo of perforated diverticulum (D) after Kocher maneuver is performed.

70 years old female was admitted to the hospital with epigastric pain, fevers and elevated white cell count. Abdominal CT scan demonstrated evidence of duodenal diverticulitis and she was started on broad-spectrum IV antibiotics (Fig. 1). Overnight, her clinical condition had worsened with persistent tachycardia, increase in white count, fevers and signs of peritonitis on exam. Interval CT revealed significant amount of air and fluid in the abdomen concerning for free perforation. Patient was consented for exploration and possible pancreatoduodenectomy. During surgery, large perforation of the 4 cm juxtapapillary duodenal diverticulum originating from posterior-medial wall with peritonitis was found (Fig. 2). Due to very medial location in close proximity to insertion of the ampulla, segmental resection was not possible and decision was made to proceed with pancreatoduodenectomy. Pathology confirmed perforation originating from duodenal diverticulum with no additional abnormal findings. Patient tolerated procedure without complications and was discharged home after 10-day hospital stay. She is doing well at 2 months follow up and has returned to work.

## 3 Discussion

Duodenal diverticula are quite prevalent in general population, seen on up to 5% of radiology studies and up to 22% of autopsy examinations.^2^ Only 5% of all diverticula will ever become symptomatic. Clinical presentations include diverticulitis, perforation with localized abscess, fistulization, duodenal and bilio-pancreatic obstruction and bleeding [2]. Endoscopic therapy is the main therapeutic tool for diagnosis and management of certain complications such as bleeding and pancreatic or biliary obstruction [[2], [3], [4], [5]]. Conservative therapy with antibiotics and bowel rest is successful in majority cases of perforation [5]. Failure of conservative therapy could be associated with high mortality and demands surgical management. Variety of surgical approaches ranging from simple diverticulectomy to segmental resection, duodenal exclusion/bypass to pancreatoduodenectomy are available [[3], [4], [5], [6]].

## 4 Conclusion

Pancreatoduodenectomy is very rarely performed in situation of acute perforation but may be used when all other approaches are limited due to perforation location and inability to carry out local/segmental resection [5].

Authors have no conflicts of interest to declare.

No specific funding was used in preparation of this article.

## Conflicts of interest

Authors have no conflicts of interest to declare.

## Sources of funding

No specific funding was used in preparation of this article.

## Ethical approval

Exception from ethical approval – case report only, consent from patient provided at request.

## Consent

Written informed consent was obtained from the patient for publication of this case report and accompanying images. A copy of the written consent is available for review by the Editor-in-Chief of this journal on request.

## Author contribution

Justus Philip: conceptualisation, methodology, software, validation, formal analysis, investigation, resources, data curation, writing – original draft, writing – review and editing, visualisation, project administration, funding acquisition.

Andrei Cocieru: conceptualisation, methodology, writing – review and editing, supervision.

## Registration of research studies

Not required.

## Guarantor

Andrei Cocieru, MD.

## Provenance and peer review

Not commissioned, externally peer-reviewed
